# Proximity of Wildfires to Inpatient Healthcare Facilities in California, 2001–2023

**DOI:** 10.1029/2025GH001531

**Published:** 2026-04-03

**Authors:** Caleb Dresser, Neil Singh Bedi, Andrew Schroeder, Eric Sergienko, Satchit Balsari

**Affiliations:** ^1^ Department of Emergency Medicine Beth Israel Deaconess Medical Center Boston MA USA; ^2^ Harvard Center for Climate, Health, and the Global Environment Department of Environmental Health Harvard TH Chan School of Public Health Boston MA USA; ^3^ Harvard T.H. Chan School of Public Health Boston MA USA; ^4^ Boston University Chobanian and Avedisian School of Medicine Boston MA USA; ^5^ Direct Relief Santa Barbara CA USA; ^6^ Former Health Officer Mariposa County Health and Human Service Agency Mariposa CA USA; ^7^ Department of Global Health and Population Harvard T.H. Chan School of Public Health Boston MA USA

**Keywords:** wildfire, hospital, spatial, disaster, preparedness, climate

## Abstract

This study assessed changes in the proximity of wildfires to inpatient healthcare facilities in California during the period 2001 to 2023. This retrospective, descriptive spatial analysis of 22 years of wildfire perimeter and healthcare facility data analyzed distances between each inpatient facility and the nearest wildfire perimeter in each year (wildfire‐facility distance). Distances were computed on an annual basis using data from the California Department of Health Care Access and Information and CAL‐FIRE's Fire and Resource Assessment Program. Temporal changes in wildfire‐facility distances over the study timeframe were analyzed via linear modeling and Kruskal‐Wallis test. This analysis revealed that distances from inpatient healthcare facilities in California to nearby wildfires are decreasing by an average of 628 feet per year. More facilities are experiencing nearby wildfires. During 2017–2023, there were 53% more inpatient beds within five miles of a wildfire than in 2001–2008. Wildfires are occurring closer to inpatient healthcare facilities in California. An increasing proportion of California's inpatient bed capacity is exposed to nearby wildfires. Policies to reduce risk posed by wildfires, prepare for evacuations, preserve access to healthcare, and ensure safe location of new facilities are urgently needed to ensure the safety of patients and the wellbeing of populations that depend on inpatient healthcare services.

## Plain Language Summary

Wildfires can cause damage or evacuation of hospitals and other inpatient healthcare facilities. In this study, we analyzed 22 years of data on where wildfires occurred in California in relation to these healthcare facilities. We measured how close the nearest fire was to each facility in each year, and compared changes in these distances over time. Our analysis shows that these nearest wildfires have been getting closer to healthcare facilities by an average of 628 feet per year, and that the number of inpatient beds exposed to a wildfire within a five mile radius has increased by 53% in the most recent third of the data set as compared with the earliest third. We also found that recently built healthcare facilities, particularly nursing homes, have been experiencing a disproportionate level of exposure to nearby wildfires. These findings suggest the need for increased attention to the safe siting of new facilities, readiness of facilities for evacuations, and other risk reduction actions.

## Key Points

- Wildfires are occurring closer to healthcare facilities in California over the past two decades
- Recently constructed facilities experienced a disproportionate number of close approaches during recent wildfires
- Risk reduction approaches need to be developed for existing facilities, and siting of new facilities needs to be carefully considered in the context of this hazard

## 1 Introduction

Wildfires in California, USA are becoming larger and more destructive (EPA, 2016). Worsening wildfire risks are related to factors including changing development and land use patterns, human activity and related ignition sources, and the impacts of global climate change (Radeloff et al., 2018). Of the 20 largest fires in state history, 13 took place in 2015 or later (CAL FIRE Statistics, n.d.). Fifteen of the 20 most destructive wildfires took place in the same time period (CAL FIRE Statistics, n.d.).

Wildfires and their smoke affect human health through both direct and indirect pathways. In addition to causing respiratory illnesses, impacts of wildfire smoke on patients with cardiovascular or respiratory disease and other conditions are also well documented (Aguilera et al., 2021; Dohrenwend et al., 2013; Finlay et al., 2012; Reid et al., 2016). Evacuations, property damage, and disruptions in critical infrastructure, social functioning and economic activity have also been shown to result in negative impacts on health (Hamideh et al., 2022; Rosenthal et al., 2021; United States Joint Economic Committee, 2023). Health systems can suffer both direct damage to their structures and indirect effects related to disruption of critical infrastructure, transportation networks, supply chains, and population displacement, including staff evacuations (Fraser et al., 2022; Modaresi Rad et al., 2023).

A large proportion of the inpatient bed capacity in California is located near fire threat zones described as “high”, “very high”, or “extreme” by the California Department of Forestry and Fire Protection (CAL‐FIRE), with half of California's inpatient bed capacity located less than 0.87 miles from a high fire threat zone in 2022 (Bedi et al., 2023). However, empirical information on how this risk translates into actual exposure of healthcare facilities to wildfires, information on temporal trends in their exposure, and information on spatial distribution of such exposures are lacking. This information is important for assessing vulnerability, identifying adaptation opportunities, and prioritizing finite resources toward settings where they will be maximally beneficial.

In this descriptive analysis, we assess wildfire proximity to critical healthcare infrastructure in California, specifically inpatient healthcare facilities. This retrospective, descriptive spatial analysis provides information on the changing proximity of wildfires to inpatient healthcare facilities in California during the period 2001–2023.

## 2 Data and Methods

This study consisted of a retrospective geospatial analysis of distances from inpatient healthcare facilities in California to the nearest wildfire in each year using publicly available data on healthcare facilities and historical wildfire perimeters (Figure S1 in Supporting Information S1).

Healthcare Facility Data: Inpatient healthcare facility data (2001–2023) was sourced from the California Department of Health Care Access and Information (Department of Health Care Access and Information, n.d.). To our knowledge, the California Licensed and Certified Healthcare Facility Listing is the most complete, accurate, and official data set of operational civilian healthcare facilities in California. Veterans Care Homes classified as long‐term care facilities (LTCFs) are included in the data set; military healthcare facilities and Veterans Affairs hospitals are not included. Inpatient facilities include general acute care hospitals, psychiatric hospitals, chemical dependency recovery hospitals, skilled nursing facilities, intermediate care facilities, congregate living health facilities, and hospice facilities, comprising all facilities where patients require overnight care and could require evacuation in the event of a disaster. Facilities that had suspended licenses, were closed, or were otherwise non‐operational were excluded from analysis in the applicable years.

Wildfire Perimeter Data: Data on wildfire perimeters was retrieved from the CAL‐FIRE Fire and Resource Assessment Program (FRAP) (CAL FIRE Fire Perimeters, n.d.). Wildfire perimeter data is based on information from ground teams, aerial surveillance, and satellite imagery. Although CAL‐FIRE FRAP hosts “the most complete digital record of fire perimeters in California,” some fires may be missing, may have been excluded due to minimum cutoffs, may be insufficiently or inaccurately documented, or have not yet been added to the database (CAL FIRE Fire Perimeters, n.d.).

*Distance Calculations*: The Euclidean distance from the geocoordinates of each facility that was open in a given year to the nearest point or vector in any wildfire perimeter from that year was computed using the st_distance function in the sf package in R; this process was repeated separately for each year in which the facility was open (Pebesma & Bivand, 2023). Geospatial data in early years of the data set was primarily in the form of street addresses; healthcare data sets for later years included geocoordinates. For facilities for which geocoordinate data was not provided, the GoogleMaps API was used to compute geocoordinates, and any facilities that were not geocoded successfully by this processing method were geocoded manually on GoogleMaps. Orthodromic distance calculations were not utilized given the local nature of the analysis and short distances being assessed. A single numerical value representing the Euclidean distance from the facility to the nearest wildfire perimeter (“wildfire‐facility distance”; Figure S2 in Supporting Information S1) was computed for each facility for each year. In cases where facilities were within the fire perimeter, distances were coded as zero.

*Analysis*: A simple linear regression was performed, taking wildfire‐facility distances as a response variable and year as a predictor variable. The purpose of this regression was to assess the overall trend in distances from facilities to wildfires over time; causal inference was not attempted in this descriptive analysis. A Kolmogorov‐Smirnov test was performed to assess the normality of the distribution of the distances in each year and was consistent with a nonnormal distribution; Figure S3 in Supporting Information S1 provides a graphical depiction of the distribution of distances in each year. The data set was subdivided into three time periods (tertiles) by year, specifically 2001–2008, 2009–2016, and 2017–2023. These periods were chosen to produce tertiles of even size, and the cut‐point years do not correspond to external policy or environmental conditions or events. Differences in distances from facilities to wildfires were assessed by Kruskal Wallis tests, due to the nonnormal distribution of wildfire distances within each year. A Dunn test was conducted, and differences of medians were computed between each pairing of the three tertiles. Cumulative inpatient bed capacity percentage plots relative to distance to nearest wildfire were constructed for the three time periods.

A sub‐analysis of the late study period (2017–2023) was performed to assess whether more recently opened facilities were disproportionately contributing to the lower wildfire‐facility distances in the late period; a Wilcoxon rank sum test was conducted to compare wildfire‐facility distances of facilities that were first licensed and opened in or after 2017 against those licensed prior to 2017. Wildfire‐facility distances were also disaggregated by year of facility opening to allow graphic representation of relationships between wildfire‐facility distances, passage of time, and year of licensure of facilities.

For each of the three time periods, we identified the total number of wildfire‐facility distances that were ≤5 miles and aggregated them to the county level. We also computed the proportion of all wildfire‐facility distances that were ≤5 miles (out of all wildfire‐facility distances) for each county. Using these data, we created a panel of bivariate choropleths that highlight the spatial and temporal variation in wildfire exposure to facilities at the county level. This method identifies counties in which a large absolute number of wildfire‐facility distances were ≤5 miles as well as counties with a high proportion of ≤5 mile events relative to the total number of facilities in the county, identifies counties in which these metrics of exposure intersect, and allows assessment of changes in the spatial distribution of these exposures over time.

All analyses were completed using R (Version 4.3.1) in RStudio (Version 2024.04.2 + 764) and Microsoft Excel (Version 16.86). This study utilized publicly available data from the State of California, did not involve human subjects research, and did not require IRB review. The analysis code is available in a public GitHub repository (https://github.com/nsbedi/wildfire‐distances‐inpt‐hcfs).

This study utilized publicly available data from the State of California, did not involve human subjects research, and did not require IRB review.

## 3 Results

The annual number of inpatient healthcare facilities ranged from 1,588 in 2002 to 1,867 in 2020, with a modest upward trend in the total number of facilities during the study timeframe (Table 1). Inpatient healthcare facilities included general and acute care hospitals and inpatient psychiatric hospitals (“hospitals”), which accounted for 492 to 518 facilities per year, as well as LTCFs , which accounted for 1,070 to 1,367 facilities per year. Growth in the total number of facilities over time was predominantly due to the increasing number of LTCFs (Table 1).

**Table 1:** Inpatient Healthcare Facilities and Wildfires in California, 2001‐2023

Year	Hospitals	LTCFs	Total facilities	Number of fires^a^	Acres Burned^a^ ^,^ ^b^	Median wildfire‐facility distance (miles)
2001	512	1,202	1,714	206	246,925	17.24
2002	518	1,070	1,588	243	963,899	12.27
2003	509	1,135	1,644	339	968,697	14.12
2004	501	1,194	1,695	277	274,639	14.94
2005	500	1,180	1,680	304	231,112	14.19
2006	502	1,188	1,690	314	748,170	14.61
2007	503	1,187	1,690	348	1,031,803	9.5
2008	502	1,189	1,691	439	1,382,710	10.64
2009	499	1,192	1,691	254	435,825	12.14
2010	501	1,191	1,692	207	101,056	14.31
2011	502	1,193	1,695	316	202,423	14.44
2012	500	1,199	1,699	352	848,692	12.78
2013	498	1,200	1,698	297	569,422	14.42
2014	500	1,215	1,715	236	554,656	16.42
2015	497	1,248	1,745	317	789,222	10.6
2016	497	1,266	1,763	361	546,250	11.73
2017	492	1,324	1,816	608	1,424,583	7.69
2018	497	1,338	1,835	416	1,581,917	10.5
2019	493	1,353	1,846	319	280,728	9.75
2020	493	1,374	1,867	505	4,182,335	8.83
2021	496	1,365	1,861	388	2,512,611	9.85
2022	498	1,367	1,865	306	322,528	10.06
2023	500	1,347	1,847	284	342,033	18.21

^a^ Data sourced from CAL‐FIRE FRAP.

^b^ Rounded to the nearest whole acre.

In the period 2001–2023, CAL‐FIRE FRAP recorded 22,261 fires, with annual burned acreage ranging from 101,056 to 4,182,335 acres (Table 1). There was an increase in the number of acres burned per year during the study timeframe; four of the six years in which more than 1,000,000 acres burned occurred in the second half of the years in the analysis period.

A total of 40,027 wildfire‐facility distances were computed over the 23‐year study timeframe. The result was a single data set of operational inpatient healthcare facilities in California in each year (2001–2023) and the distance from each facility to the nearest wildfire perimeter recorded by CAL‐FIRE that year. This data set has been made publicly available in a data repository (Bedi & Dresser, 2025).

### 3.1 Distribution of Wildfire‐Facility Distances

**Figure 1:**
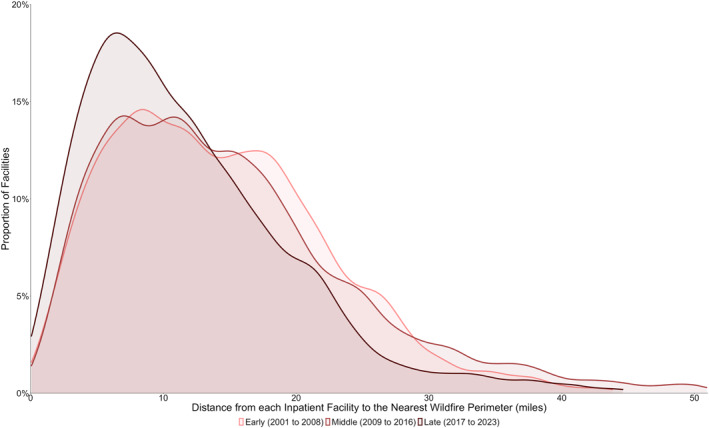
Wildfire‐Facility Distance Distributions in the Early, Middle, and Late Time Periods. Distribution of wildfire‐facility distances for the Early (2001–2008), Middle (2009–2016), and Late (2017–2023) time periods. Wildfires were closer to healthcare facilities in the late period as compared with the early and middle time periods.

Distances from inpatient healthcare facilities to the nearest wildfire perimeter ranged from 0 to 167.7 miles; annual median wildfire‐facility distances ranged from 7.69 to 18.21 miles (Table 1). Wildfire‐facility distances in each year were asymmetrically distributed with a rightward skew, reflecting the subset of facilities that did not experience any nearby fires in that year (Figure 1). The data was non‐normally distributed in all years, as assessed by the Kolmogorov‐Smirnov test (*P* < 0.001 for all years); the shape of the distributions was relatively consistent across years (Figure S3 in Supporting Information S1).

### 3.2 Temporal Changes in Wildfire Facility Distances

A univariate linear model was constructed to assess the relationship between wildfire‐facility distances and time. This model incorporated distances as a response variable and year as a predictor variable; no attempt was made to adjust for covarying factors, as the goal was to describe the trend in observed data rather than make causal inferences. In this descriptive model, the passage of time was associated with a statistically significant reduction in wildfire‐facility approach distances of approximately 0.128 miles (678 feet; 206 m) per year (*P* < 0.0001). The overall explanatory value of the model for this system was poor (*R* ^2^ = 0.008), as was expected given the univariate approach, the stochasticity of wildfire location each year, and the decision to use a descriptive rather than causal or predictive modeling approach.

To further assess the implications of changes in wildfire‐facility distances over time, the data set was subdivided into temporal tertiles: “early” (2001–2008), “middle” (2009–2016), and “late” (2017–2023). Kruskal‐Wallis confirmed a statistically significant difference in wildfire‐facility approach distances between time periods (*H* = 952.8, df = 2, *P* < 0.0001), and a Dunn test confirmed the significance of differences between individual subsets (*P* < 0.0001 for late‐early and late–middle). There were lower median wildfire‐facility distances in the late period (10.32 miles, IQR 10.12) as compared with the early (13.33 miles, IQR 11.55) and middle (13.27 miles, IQR 12.20) time periods. A larger proportion of inpatient healthcare facilities experienced nearby wildfires in the late period as compared with the early and middle time periods (Figure 1).

**Figure 2:**
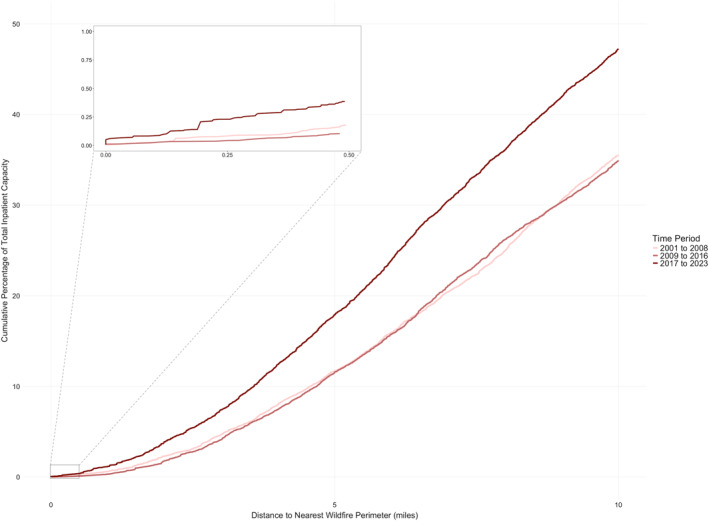
Cumulative Percentage of Total Inpatient Bed Capacity Exposed to Nearby Wildfires in the Early, Middle, and Late Time Periods. Total inpatient bed capacity was aggregated for the Early (2001–2008), Middle (2009–2016), and Late (2017–2023) time periods based on proximity to the nearest wildfire perimeter and is presented via cumulative percentage plots. The proportion of inpatient healthcare capacity within five miles of a wildfire approach was 53% higher in the most recent (Late) period as compared to previous time periods.

Total inpatient bed capacity for each of the three time periods was aggregated by proximity to the nearest wildfire perimeter and is presented in cumulative percentage plots (Figure 2). The proportion of inpatient bed capacity that experienced the occurrence of a wildfire within five miles was 53% higher in the late period (2017–2023) than in the early period (2001–2008). Change in exposure in the late period relative to the middle period showed a similar increase (Figure 2).

### 3.3 Wildfire Proximity to Recently Opened Inpatient Healthcare Facilities

Over the course of the study period, 486 new inpatient facilities opened, of which 128 were hospitals and 358 were LTCFs. An analysis was performed to assess whether the opening of new healthcare facilities (which may have been built in higher risk locations) contributed to the observed decrease in wildfire‐facility distances in the late period (2017–2023) described above. Wildfire‐facility distances for years 2017–2023 were separated into those representing facilities opened prior to 2017, and those opened in 2017 or later. Due to the skewed, non‐normal nature of the data, wildfire‐facility distances in these groups were compared via Wilcoxon rank sum test; the median wildfire‐facility distance for new facilities (those first licensed and opened in 2017 or later; median 8.86 miles, IQR 7.59) was lower than for older facilities (median 12.36 miles, IQR 11.57) by 3.50 miles (*p* < 0.0001).

**Figure 3:**
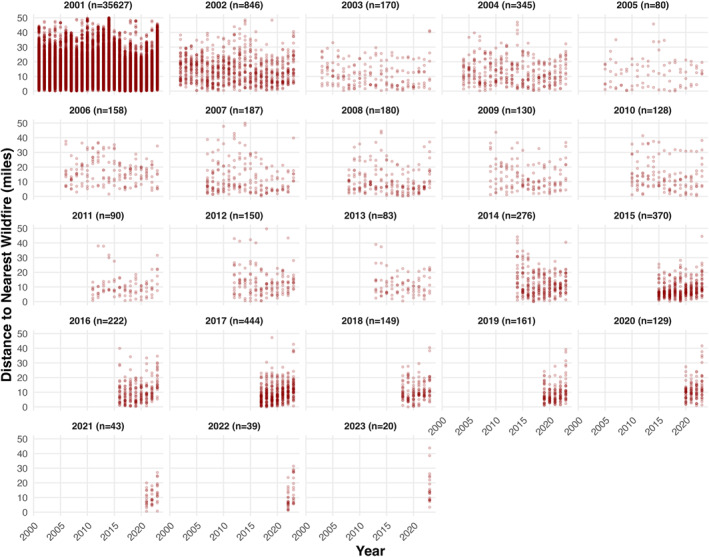
Wildfire proximity to inpatient healthcare facilities, stratified by facility year of opening. Each panel displays distances from each facility to the nearest wildfire perimeter, grouped by the year in which facilities first appear in the overall data set. Each point represents an individual wildfire‐facility distance. Most wildfire‐facility distances (35,627 of the 40,027) are in the 2001 panel, representing facilities that were opened prior to or during 2001. Lower wildfire‐facility distances are seen in later years for facilities opened earlier in the study period; facilities opened late in the study period tend to exhibit lower wildfire‐facility distances compared with older facilities. Note: the *y*‐axis is truncated at 50 miles for easier interpretation; of 40,027 data points, 249 (0.62%) are excluded from this figure because wildfire‐distance exceeded 50 miles.

To assess whether the previously described decrease in wildfire‐facility distances over time was related to other factors in addition to the opening of new facilities, the simple linear modeling approach described above was applied to a subset of the overall study data consisting of wildfire‐facility distances for facilities that were already open at the start of the study timeframe (2001). In this model, which excluded facilities that were opened during the study period and thus excludes the effect of any facilities that were newly opened in high‐risk locations, the passage of time was associated with a reduction in wildfire‐facility approach distances of approximately 0.103 miles (543 feet; 166 m) per year (*p* < 0.0001). Plots of wildfire‐facility distances disaggregated by year of facility opening are presented in Figure 3.

### 3.4 Spatial Distribution of Wildfire Exposure at Inpatient Healthcare Facilities

The spatial distribution of facilities, wildfires, and wildfire‐facility approach distances in California during the study timeframe was uneven. Inpatient healthcare facilities are concentrated in urban and peri‐urban areas, particularly along the coast and in the Central Valley, with much smaller numbers of facilities located in rural and mountainous parts of the state (Figure S1 in Supporting Information S1).

**Figure 4:**
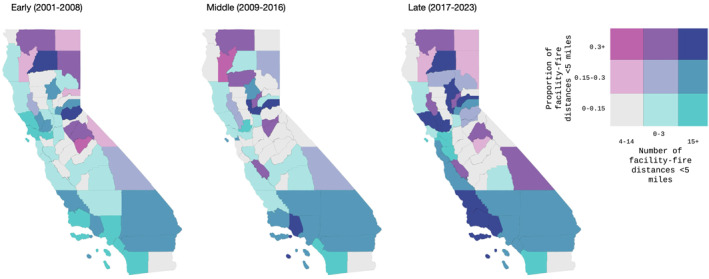
Number and Proportion of Inpatient Facilities within Five Miles of Wildfire Perimeters during the Early, Middle, and Late Time Periods. This bivariate choropleth depicts the number of facilities within each county with wildfire‐facility distances ≤5 miles (aggregated into textiles along the horizontal color palette) and the proportion of facilities within each county with wildfire‐facility distances ≤5 miles (along the vertical color palette).

The number and the proportion of facilities in each county experiencing wildfire approaches within 5 miles in the early, middle, and late study periods are presented in bivariate choropleths (Figure 4). In the counties of northern and northeastern California, there are comparatively few total inpatient healthcare facilities, but a high proportion of these facilities have experienced wildfire approaches within 5 miles. In contrast, a lower proportion of the inpatient healthcare facilities in counties in coastal and southern California experienced nearby wildfires, but the very large total number of facilities in these counties meant that the total number facilities threatened by wildfires within 5 miles of their location in these counties was higher than in more rural areas. The proportion of facilities and total number of facilities experiencing wildfire approaches within 5 miles was low in most counties of the Central Valley. Across California, the number of counties in which a high proportion of facilities and a high total number of facilities experienced nearby wildfires increased over the course of the study period (Figure 4, dark purple).

## 4 Discussion

Wildfire disasters in California are worsening; inpatient healthcare facility sites are increasingly exposed to this hazard. Utilizing annual wildfire perimeter and healthcare facility location data, we show that wildfires are now occurring in closer proximity to inpatient healthcare facilities, and that an increased proportion of California's inpatient bed capacity has been exposed to nearby wildfires in recent years. Wildfire exposure distribution is uneven across California's counties; in rural counties in northern and eastern California, a high proportion of facilities have experienced nearby wildfires, while in densely populated coastal areas, a larger total number of facilities have experienced nearby wildfires; absolute and proportional exposure to nearby wildfires has been low in some areas, including the Central Valley.

Throughout the study period, some facilities opened, closed, or moved; the number of LTCFs increased substantially. Analysis of recent wildfire‐facility distances (2017–2023) reveals that recently licensed facilities (opened in or after 2017) were substantially more exposed than older facilities, with the median wildfire‐facility distance for recently opened facilities being 3.5 miles closer than for older facilities during recent wildfires. However, among facilities that were present in the first year of the data set (2001), decreases in wildfire facility distances were also observed over the course of the study timeframe; this increase in exposure is not attributable to the opening of new facilities during the study time frame.

Attribution of observed changes to specific causes other than the opening of new facilities was not attempted in this study, which focuses on presenting overall trends in healthcare facility hazard exposure, informing decisions about the siting of new healthcare facilities, and informing the prioritization of resources for wildfire preparedness at healthcare facilities. The observed decrease in the distances between inpatient healthcare facilities and wildfires, which remains present even when new facilities are excluded, is likely multifactorial (Balch et al., 2024; Modaresi Rad et al., 2023; Radeloff et al., 2018). Development and demographic change in the wildland‐urban interface and changes in regional climate, wildland fuels management, firefighting capacity or strategy, and ignition sources are potential contributors which could be topics for future analyses of the data set assembled for this project, which is available in a public repository (Bedi & Dresser, 2025).

### 4.1 Limitations

Wildfires are complex events with inherently stochastic elements. This study describes overall patterns in a large retrospective data set, but cannot predict specific local risks; the absence of nearby wildfires in a specific location does not mean they cannot occur. Resources including CAL‐FIRE's fire threat assessment maps are designed to provide a portrait of the spatial distribution of future wildfire risk, and provide an alternative means to assess risk to specific health facilities (Bedi et al., 2023).

The data sets utilized in this analysis provide information that is internally comparable state‐wide and over time with respect to methodology and data structure. However, limitations with respect to both facility and fire data sets are important to understand when interpreting our findings. With respect to wildfire perimeters, some wildfires may be missing, may have been excluded, or may be inaccurately documented in the CAL‐FIRE FRAP database (CAL FIRE Fire Perimeters, n.d.). For facilities, it is important to note that Veterans Affairs hospitals and military facilities were not included in the database utilized in this analysis.

The trends in wildfire‐facility distances identified via our descriptive linear models must be interpreted with caution. Changes in future fuel availability, fire weather, ignition sources, wildland management, and human settlement and facility construction patterns may be nonlinear; extrapolation of results from this descriptive study into the future may not have predictive value. We did not attempt to assess the influence of or control for potential causal or confounding factors such as climate forcing, El Nino/La Nina, demographic trends, socioeconomic trends, policy changes, or the effects of the COVID‐19 pandemic in this descriptive analysis. As such, our results should be interpreted as a portrait of recent wildfire exposure; additional research will be needed to understand the causes of the trends we describe and to assess how risk to healthcare facilities posed by wildfires may evolve in future years.

### 4.2 Policy Implications

Addressing the threat to healthcare facilities will require actions at multiple levels, including individual facilities, healthcare systems, and governmental policy (Hertelendy et al., 2024). Goals include reducing physical risk to facilities, preparing for evacuations, and ensuring access to care.

Reducing physical risk to existing facilities involves hardening healthcare facilities and actions to reduce fire risk in the surrounding landscape. While federal guidance exists and state law requires creating defensible space around structures in fire‐prone areas, funding for capital investments in fireproofing hospital grounds and buildings is limited and represents a policy opportunity (California Code, PRC 4291, n.d.; Natural Disasters, n.d.).

Reducing risk to new facilities requires consideration of fire threat during the siting, permitting, design, and construction process. A large fraction of the increase in wildfire exposure identified in this study is linked to newly opened inpatient healthcare facilities, predominantly LTCFs. Policies to ensure safe siting of future LTCFs and design and construction practices that prioritize fire safety can help California avoid investing in new healthcare infrastructure that suffers from inherent long‐term risks related due to wildfire exposure.

Wildfires create complex evacuation challenges. Acute care hospitals and LTCFs must safely transfer patients who may be unstable or ventilator dependent. Inpatient psychiatric facilities must maintain direct observation of involuntarily hospitalized patients who may have behavioral instability or be at elevated risk of suicidal behavior. Rural facilities face lengthy transfer times that reduce evacuation efficiency. Safe, timely evacuation of inpatient healthcare facilities requires EMS assets, availability of receiving sites, and management of resources across multiple institutions and agencies. Coordination of requests from affected facilities can help address competition for ambulances and other resources. Dedicated ambulance strike teams can augment local EMS assets (Ambulance Strike Team, n.d.). Hospital checklists can support decision‐making and evacuation (Obata, 2024). Tabletop exercises, drills, investment in agencies capable of coordinating large‐scale inter‐facility transfers, and planning for the needs of medically vulnerable populations using resources such as HHS's Empower data set can also support readiness (Balsari et al., 2021).

Wildfire severity is projected to continue to rise in future years (Wasserman & Mueller, 2023). Strategies to address the increasing risk of wildfires to healthcare infrastructure are urgently needed to protect healthcare access and ensure the well‐being of individuals impacted by these events. Additional research utilizing the data set we have shared above, in conjunction with climate, demographic, and other data sources, may be helpful in developing causal models and/or projections of future wildfire exposure risk; parallel high resolution geospatial research is also needed to understand and project future risk to specific health facilities. Policies to address the root causes of the increase in wildfire exposure are also essential. It is critical that health professionals and policymakers address this imminent threat to critical healthcare infrastructure; lack of proactive efforts may lead to devastating consequences.

## 5 Conclusion

California is experiencing increasingly severe wildfires and has an increasing number of inpatient healthcare facilities. Distances from inpatient healthcare facilities to nearby wildfires have decreased over the past 23 years, and an increasing proportion of inpatient beds are exposed to wildfire approaches within 5 miles of their facility. Policies to address this risk are urgently needed to protect the wellbeing of the populations served by these health systems.

## Conflict of Interest

The authors received grants from Biogen and Johnson and Johnson outside the submitted work.

## Supporting information

Supporting Information S1

## Data Availability

The data set assembled for this project is available in a public repository (Bedi & Dresser, 2025). The analysis code is available in a public Zenodo repository (nsbedi & calebdresser, 2025).
