# Supplementary material for: Proximity of Wildfires to Inpatient Healthcare Facilities in California, 2001–2023
Source: Geohealth. 2026 Apr 3;10(4):e2025GH001531. doi: 10.1029/2025GH001531 (PMC13052163; doi:10.1029/2025GH001531)
Supplement: Supplementary file 1 — Supporting Information S1 [file GH2-10-e2025GH001531-s001.pdf]

Proximity of Wildfires to Inpatient Healthcare Facilities in California, 2001-2023

Authors

Caleb Dresser MD MPH[1,2], Neil Singh Bedi MPH[3,4], Andrew Schroeder PhD MPP[5], Eric Sergienko MD MPH[6], Satchit Balsari MD MPH[1,7]

1. Department of Emergency Medicine, Beth Israel Deaconess Medical Center, Boston, MA
2. Harvard Center for Climate, Health, and the Global Environment, Department of Environmental Health, Harvard TH Chan School of Public Health, Boston, MA
3. Harvard T.H. Chan School of Public Health, Boston, MA
4. Boston University Chobanian and Avedisian School of Medicine, Boston, MA
5. Direct Relief, Santa Barbara, USA
6. Former Health Officer, Mariposa County Health and Human Service Agency
7. Department of Global Health and Population, Harvard T.H. Chan School of Public Health

Table of Contents

Page	Figure
2	Figure S1: Inpatient Healthcare Facilities and Wildfires 2001 – 2023.
3	Figure S2: Methods Explanation: Case Series of Butte County
4	Figure S3: Ridgeline Plot Depicting the Annual Wildfire-Facility Distance for Inpatient Healthcare Facilities in California (2001-2023).

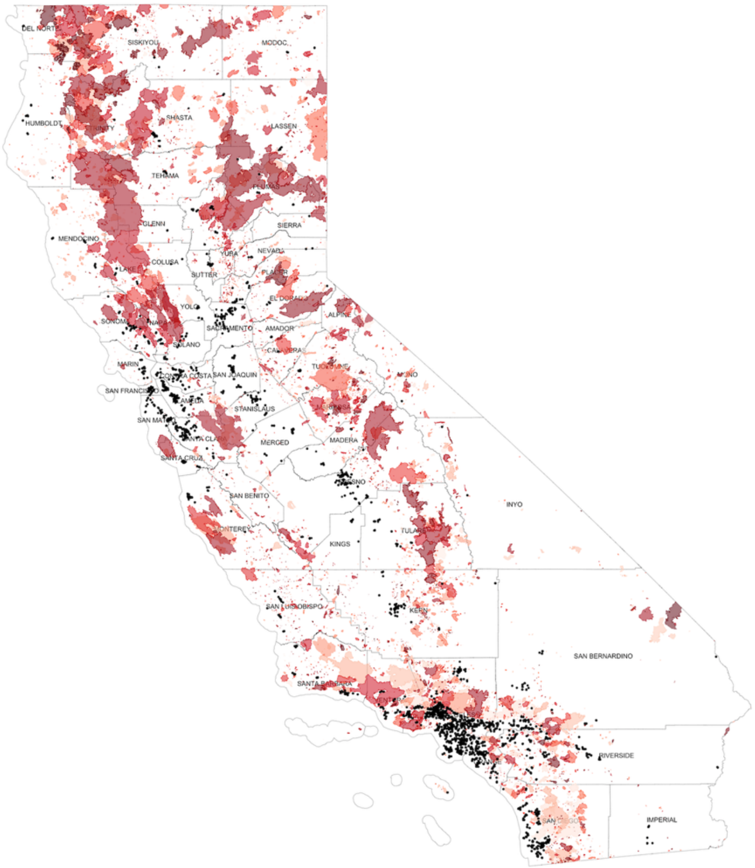

Figure S1: Inpatient Healthcare Facilities and Wildfires 2001 – 2023. All wildfires and inpatient facility locations used for analysis, plotted within the State of California. The polygon shading represents the year in which the fires occurred; darker shading represents more recent years. Black dots represent locations of inpatient facilities.

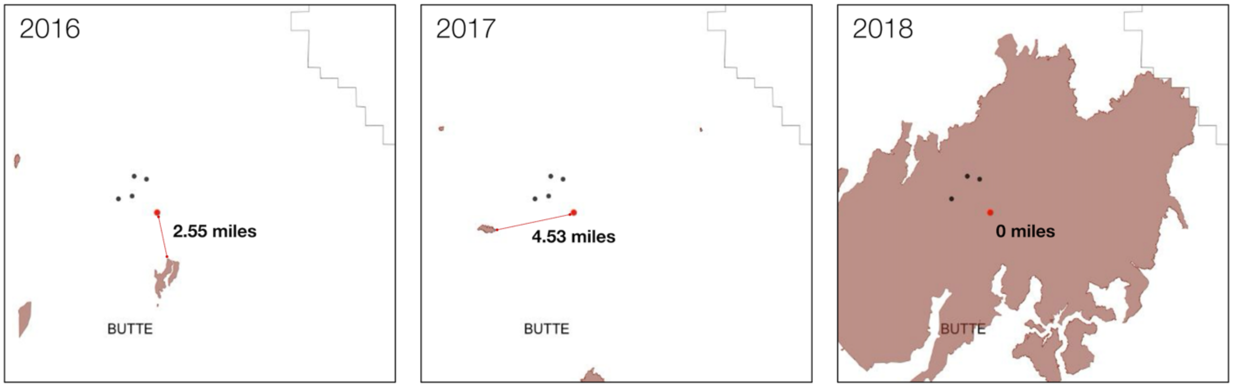

Figure S2: Methods Explanation: Case Series of Butte County. The panels represent three years (left, 2016; middle, 2017; right 2018) of wildfire occurrences and inpatient facility locations within Butte County. The red dot in each panel represents Adventist Health Feather River Hospital; a red line shows the distance to the nearest wildfire perimeter in each year. In 2018, the Camp Fire devastated Paradise, California, and with it, Adventist Health Feather River Hospital, which was inside the wildfire perimeter; hence, its distance for 2018 was computed as ‘0’.

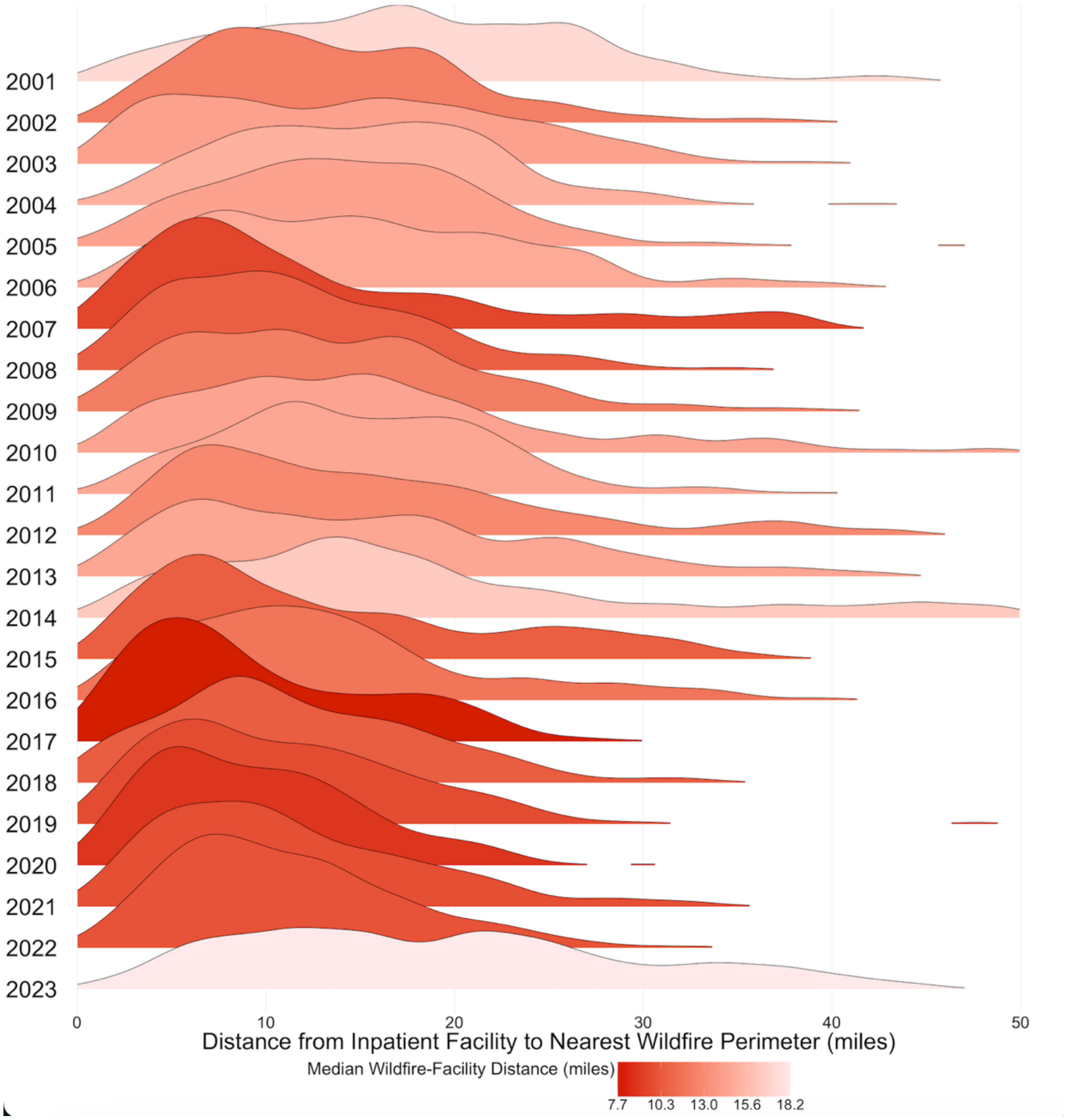

Figure S3: Ridgeline Plot Depicting the Annual Wildfire-Facility Distance for Inpatient Healthcare Facilities in California (2001-2023). Each distribution ridge represents the wildfire-facility distance distribution in that year. The ridges are shaded based on the median distance to the nearest wildfire perimeter; a lower median distance is indicated by a darker shade of red.
